# CAREGIVER EXPERIENCE WITH FRONTOTEMPORAL DEGENERATION (FTD)

**DOI:** 10.1093/geroni/igad104.2545

**Published:** 2023-12-21

**Authors:** Esther Kane, Shana Dodge, Sweatha Reddy, Penny Dacks

**Affiliations:** AFTD, King of Prussia, Pennsylvania, United States; The Association for Frontotemporal Degeneration, King of Prussia, Pennsylvania, United States; FTD Disorders Registry, King of Prussia, Pennsylvania, United States; AFTD, King of Prussia, Pennsylvania, United States

## Abstract

Frontotemporal degeneration (FTD) is the umbrella term for a group of young onset neurodegenerative disorders affecting the frontal and temporal lobes of the brain. Symptoms include changes in cognition, language, behavior, motor functioning, and personality. The FTD Insights Survey was designed to learn more about the lived experience of this often misunderstood set of disorders. A total of 1,246 past and current caregivers of people with FTD responded to questions regarding their and their loved one’s experiences with FTD. Caregivers most often provided care for more than 80 hours per week and 10% of respondents provided care for more than one person diagnosed with FTD. The majority were caring for someone or had cared for someone who was diagnosed between ages 50 and 69. Only 36% of caregivers reported the person diagnosed was aware or mostly aware of their symptoms and they noted being most distressed by changes in the diagnosed person’s language, personality, mood, and behavior. The caregivers described the impact of their loved one’s symptoms on activities of daily living at home, in the community, and on interpersonal functioning. The caregivers also provided insight into issues such as the length of time they have provided care, the level of functioning of the person diagnosed, and the impact of FTD symptoms on quality of life. It is critical that healthcare professionals and policy makers understand FTD caregiver strain in order to provide wraparound support and make policy decisions with these features in mind.

